# Change in the Prevalence and Social Patterning of First- and Second-Hand Smoking in PORTUGAL: A Repeated Cross-Sectional Study (2005 and 2014)

**DOI:** 10.3390/ijerph17103594

**Published:** 2020-05-20

**Authors:** Joana Alves, Rita Filipe, João Machado, Baltazar Nunes, Julian Perelman

**Affiliations:** 1 NOVA National School of Public Health, Public Health Research Centre, Universidade NOVA de Lisboa, 1600-560 Lisboa, Portugal; JPerelman@ensp.unl.pt; 2 ACES Lisboa Ocidental e Oeiras—Public Health Unit, 2780-163 Oeiras, Portugal; rita.filipe@arslvt.min-saude.pt; 3 Department of Epidemiology, Instituto Nacional de Saúde Dr. Ricardo Jorge, 1649-016 Lisboa, Portugal; joao.p.machado@arslvt.min-saude.pt (J.M.); bnunes@ensp.unl.pt (B.N.)

**Keywords:** Portugal, smoking, second-hand smoking, socioeconomic inequalities

## Abstract

Between 2005 and 2007, important reinforcements of the tobacco legislation have been implemented in Portugal, which may have affected smoking patterns. The aim of this study was to measure the change in prevalence of first- and second-hand smoking (SHS) among adults, and its socio-demographic patterning in Portugal from 2005 to 2014. Data from the last two Portuguese National Health Interview Surveys (2005 and 2014) were used. The changes in daily smoking and SHS were measured using Poisson regressions, stratifying by sex and survey year. The inequalities were measured using relative inequality indexes (RII). From 2005 to 2014, there was a reduction in SHS (75%–54% among men, and 52%–38% among women), and a reduction in smoking among men (27%–26%), and an increase among women (9%–12%). SHS reduction was more marked among less privileged people. Among Portuguese men, inequalities in daily smoking have increased slightly, while among women the gap favoring low-educated reduced. Between 2005 and 2014, SHS decreased, but not daily smoking, particularly among women. Additionally, socioeconomic inequalities in smoking increased. Future policies should simultaneously tackle smoking and SHS prevalence, and their socioeconomic patterning. More comprehensive policies such as comprehensive national (non-partial) bans, combined with price increases could be more effective.

## 1. Introduction

Altogether, first- and secondhand smoking are the second highest risk factors contributing to the global burden of disease in many cases being responsible for 156,838 disability-adjusted life years [1]. However, the epidemiological burden is not equally distributed in the population. Premature death is more likely among lower social classes, and smoking largely contributes to it. A study from 2006 in England and Wales, Poland, and North America at the ages of 35–69 years found that the mortality rate in the lowest social strata was twice the mortality in the highest social strata [2]. This same study estimated that smoking-attributed mortality was responsible for more than half of those differences.

Evidence on tobacco control policies (TCP) among adults showed that several measures, such as tax increases, smoke-free legislation, high-intensity media campaigns, stronger advertising bans, and health warnings, comprehensive cessation treatment, and youth access laws, can reduce smoking prevalence and deaths due to smoking [3], and that smoking bans have the power to reduce exposure to second-hand smoking (SHS) [4]. However, non-targeted interventions at population level may ultimately widen socioeconomic (SE) inequalities in smoking [5]. For example, cessation programs that do not target low SE level persons might have a harmful effect on inequalities, once high SE will benefit more from cessation opportunities than low SE status persons [6]. In the same line, the literature shows that TCP have an effect on SHS [4], but the effects on inequalities are still inconclusive [6].

In Portugal, important reinforcements of the TCP have been implemented over the 2005–2007 period, which may have affected the Portuguese smoking patterns. In 2005, the Portuguese government approved the World Health Organization Framework Convention on Tobacco Control (WHO FCTC) ( Ministério dos Negócios Estrangeiros (2005). Decreto n.º 25-A/2005. Diário da República n.º 214, SÉRIE I-A, 8 de novembro); and in 2007, a new legislation was enacted mainly to protect individuals against involuntary tobacco exposure and to reduce the demand for cigarettes, in particular regarding dependence and smoking cessation ( Assembleia da República (2007). Lei n.º 37/2007, Diário da República n. 156, SÉRIE I, 14 de Agosto.). This legislation also reinforced regulation about cigarettes composition and labels, banned advertising, and prohibited sales to minors. The law included a partial smoking ban in bars and restaurants, leading to an improvement of the Portuguese position in the Tobacco Control Scale (TCS) ranking by four places [7].

Studying SHS is extremely important since it is an externality affecting people surrounding smokers due to the burning of tobacco. It contains a mixture of compounds that were found to be to be carcinogenic on subjects of a previous studies [4]. The SHS in Portugal was evaluated mainly among children [8,9], for specific outcomes [10] or among small study samples [11].

The Portuguese case is relevant because of its still relatively high smoking prevalence, namely among men (23.5% daily smoking among men according to OCDE [12]), and because last data showed that, during the 1987–2006 period, smoking was more concentrated among low SE status men, but still more prevalent among high SE women [13], indicating that Portugal was at an earlier stage of the tobacco epidemics. Thus, the aim of this study was to measure the change in prevalence of daily smoking and SHS exposure among adults, and its socio-demographic patterning, before and after implementation of tobacco control legislation (2005–2007).

## 2. Materials and Methods

### 2.1. Sample

We used data from the last two Portuguese National Health Interview Surveys, carried out in 2005/06 and 2014 [14,15]. These surveys were cross-sectional studies designed to be representative of non-institutionalized population living in Portugal. Both samples result from a multi-stage stratified probabilistic sampling scheme, were designed to represent the seven regions of the country, and included sampling weights computed as the inverse of the probability of selection in each sampling unit, adjusted for non-response and post-stratified to best represent the target population. Only individuals aged more than 25 years old were included in this analysis, in order to avoid young people who had not completed their education; in addition, individuals older than 79 years old were not considered, to reduce the selective mortality bias. Final database included 28,433 observations for 2005/06 and 15,196 observations for 2014.

### 2.2. Variables

Variables for smoking and exposure were dichotomous. Daily smoking equaled one if the participant smoked daily and zero if they did not smoke or smoked less than daily. For SHS, the question slightly changed from the 2005/06 to the 2014 survey. The question in 2005/06 was «how many time do you spend in closed spaces around smokers, throughout the week?», while in 2014, the question was «how often are you in closed spaces while other people smoke?». Thus, a dichotomous variable for SHS was created, equaling one if exposed and zero if not exposed. The participant was considered exposed when reported being always, most of time, quite amount of time, some time, or short amount of time in close spaces around smokers throughout the week in 2005/06, and when reported being daily or occasionally exposed to smoking in 2014. The participant was considered not exposed when reporting never spending time in close spaces around smokers throughout the week in 2005/06, and never been in close spaces while other people smoke in 2014.

### 2.3. Covariates

As regards SE variables, education included five categories: no education (zero years of education), primary first education (six years of education), primary education (nine years of education), secondary or post-secondary education (twelve years of education or post-secondary non-tertiary education), and tertiary education (bachelors, master or doctoral level). Income was defined as five income quintiles of family income. Family income was the household income per equivalent adult, computed with modified equivalence scale from the Organisation for Economic Co-operation and Development (OECD), which attributes different weights to different family members [16].

### 2.4. Statistical Analysis

Prevalence ratios representing the ratio between the prevalence in 2014 and in 2005-06, and respective confidence intervals, were estimated using Poisson regressions, recommended when the log-binomial has convergence problems [17]. The change in daily and SHS from 2005/06 to 2014 was computed as follows. Firstly, daily smoking was modelled using Poisson regressions [18] with education as variable of interest, adjusting for age and stratifying by sex and survey year. The same analysis was done for income. Secondly, the educational and income inequalities were measured using relative inequality indexes (RII). RII is an indicator that not only takes into account social differences in smoking but also considers the distribution of the population over the educational levels (ridit, a score that results from the midpoint of the population’s cumulative distribution in the education/income categories) [19,20]. The analyses were adjusted for age, and stratified by sex. We estimated the RII in two separated Poisson regression models for each year, and then pooled the two surveys in a third regression by including an interaction term between the ridit term and the survey year [18].

## 3. Results

Table 1 presents the descriptive statistics for the two surveys. There were 47% women in 2005, and 44% in 2014. Around 40% of the participants were 45–64 years old. The education levels changed between the two surveys, with a decrease in the percentage of individuals with no education, from 15% to 10%.

Table 2 presents the prevalence of smoking and SHS by sex, age group, education levels, and income quintiles. Among men, prevalence of daily smoking decreased from 27.16% (CI95% = [25.75;28.62]) in 2005/06 to 25.76% [24.20;27.39] in 2014. The decrease was higher among the ones with less than 44 years old, and among the highest income quintile. The prevalence of SHS decreased 21 percentage points, from 75.03% [73.60;76.40] to 53.77% [51.96;55.57]. The highest decrease was among the oldest age group, the lower educated and among the intermediate income quintiles (2nd and 3rd income quintiles). Among women, the prevalence of daily smoking increased from 9.42% [8.56;10.36] in 2005/06 to 11.79% [10.76;12.90] in 2014. The increase was higher among the 65 to 79 years old, but the prevalence was very low in these group (1.11% [0.62;1.99] in 2015/06 and 2.43% [1.57;3.74] in 2014). The increase was also higher among the low educated, and among the lowest income quintile. Among women, the prevalence of SHS decreased from 51.93% in 2005/06 to 38.39% in 2014. The decrease was highest among the oldest, the lowest educational groups, and the lowest income quintile.

The age-adjusted prevalence ratios (PR) for daily smoking and SHS are presented in Table 3. Among men, the daily smoking prevalence was higher among the men with lower education, comparing with those with tertiary education both in 2005/06 (PR = 1.46 [1.17–1.82] for the primary education) and in 2014 (PR = 1.85 [1.48–2.33] for the primary education). The PR for the first quintile of income was 1.16 [0.98–1.36] in 2005/06 and equaled 1.56 [1.28–1.90] in 2014. Among women, the PR for daily smoking among those with primary first education compared with those with tertiary education increased from 0.38 [0.29–0.50] in 2005/06 to 0.66 [0.48–0.90] in 2014, thus, it became more equal. Regarding SHS, no significant differences were observed for the PR among men in 2005/06, while the PR was significantly lower among low-education and low-income women (PR = 0.64 [0.56; 0.73]). In 2014, the PR becomes significant among men, with low-education groups being less likely to be exposed (PR = 0.90 [0.81;1.00] in 2005/06; PR = 0.78 [0.63;0.97] in 2014), while the advantage of low-educated women was reinforced (PR = 0.64 [0.56;0.73] in 2005/05; PR = 0.59 [0.46;0.75] in 2014).

Table 4 presents the RII for the age-adjusted Poisson regressions, stratified by sex and survey year. Among men, the RII for education was 1.23 [1.00–1.52] in 2005/06 and 1.74 [1.36;2.22] in 2014, showing a higher concentration of daily smoking among the low educated men in the latter year. The coefficient for the interaction between RII and survey year (1.47 [1.09;1.99]) confirms that the inequality in smoking increased unfavorably to the lower educated. For income, the RII was 1.19 [0.98–1.43] in 2005/06 and 1.81 [1.45;2.24] in 2014. The coefficient for the interaction (1.52 [1.14;2.02]) showed a significant increase in smoking inequalities. For SHS, the RII was 0.95 [0.88;1.02] but not significant in 2005/06, and in 2014 it was 0.83 [0.73–0.95], which indicates that the SHS was more concentrated among the higher educated men, and the difference increased from 2005/06 to 2014 (the interaction between RII and survey year was 0.77 [0,67;0.89]). Among women, the RII for daily smoking was 0.10 [0.06;0.13] in 2005/06 and 0.49 [0.32;0.75], showing that smoking was concentrated among the higher educated. The gap between education categories decreased (the coefficient for interaction was 6.18 [3.71;10.32]). The RII for SHS decreased slightly from 0.57 [0.50;0.64] in 2005/06 to 0.51 [0.43;0.62] in 2014(the coefficient for interaction was 0.76 [0.63;0.92]. The income-related RII for SHS showed almost no changes from 2005/06 (0.69 [0.62;0.76]) to 2014 (0.70 [0.60–0.81]), and the difference was not significant (the coefficient for interaction was 0.97 [0.81;1.16]). The analysis for SHS was repeated only among the non-smokers, as a robustness check, and the coefficients did not show any noteworthy change.

## 4. Discussion

This study showed that, in Portugal, the number of people that reported exposure to second hand smoking largely decreased, while the number of daily smokers slightly decreased among men and increased among women, from 2005/06 to 2014. Also, among Portuguese men, socioeconomic inequalities in daily smoking have increased in magnitude against the worse-off, while among women the gap reduced, due to an increase in the prevalence among less-educated subjects. SHS was large but not consistent across sub-populations, being more marked among less privileged individuals.

In most European countries smoking is more concentrated in low social status persons [21]. Southern European countries were an exception to this behavior, since the higher educated women smoked more [22]. However, in the recent past, these countries moved to a later stage in the smoking epidemic, and that behavior was considered a slight deviation from the norm [22,23]. Yet, Portuguese women still lagged behind men in what respects the above-mentioned transition. The last study, from 1987–2005/06, showed that among men the inequalities increased while among women the gap reduction was not enough to observe the expected reversal in inequalities, despite the decrease of inequalities in cessation in both sexes. This finding was observed notwithstanding the important reinforcement of TCPs [13]. Indeed, between 2005 and 2007, the Portuguese government approved the WHO FCTC and a new legislation was enacted mainly to protect individuals against involuntary tobacco exposure and to reduce the demand for cigarettes, in particular regarding dependence and smoking cessation. This legislation also reinforced regulation about cigarette composition and labels, banned advertising, and prohibited sales to minors.

In Portugal, after the introduction of the TCP, the SHS decreased but this reduction was more marked among less privileged people. We can hypothesize that the 2009 economic crisis might have changed the meaning of income quintiles, and the exclusion of people under 25 from the sample, might have left out the ones that face the higher burden of the SHS exposure. In fact, the low socioeconomic Portuguese children are exposed to high levels of SHS at home [24]. Additionally, the number of smokers slightly decreased among men but increased among women, although remaining unequally distributed. The evidence of TCP in other countries showed that, despite the positive effect of decreasing prevalence, non-comprehensive policies can harm the social distribution of smoking. Partial smoke free policies increase socioeconomic inequalities in terms of protection to SHS, while national, comprehensive smoke free policies had the power to decrease inequalities [25]. This might be the case for Portugal, where the smoking ban implemented was only partial [26]. The legislation implemented in 2005 and 2007 was not subject to inspections, for example in what concerns sells to minors, and allowed several exceptions, as for example in the case of eating and drinking establishments (like restaurants or bars) with separated non-smoking areas and air extracting systems. Due to the study design, we cannot attribute the effect to the partial ban; but we also cannot ignore that the SHS reduction was more marked among the less privileged individuals. Thus, we hypothesize that the ban made smokers move outside (or to specific smoking areas within the establishments) to smoke, reducing the involuntary exposure to tobacco smoke, but this was not enough to make them quit smoking. Similar studies were made in Spain, following a comprehensive smoke free legislation. Authors concluded that comprehensive bans decreased smoking in settings targeted for the legislation, but the reductions were also observed in other settings not covered by the legislation (as bus stops, homes, and train stations) [27,28]. Unfortunately, we do not have enough data to investigate those effects.

This study also has some limitations. The question for SHS changed from 2005/06 survey to 2014 survey. However, we computed a dichotomous variable for exposure to SHS (high versus low), in order to minimize the impact of this change. Also, in Portugal, the existent surveys do not allow to build a trend to infer the real impact of recent legislation on smoking and SHS. In this period there were other changes in the legislation in place that might interfere with the effect (for example a ban to the sale of tobacco products to individuals younger than 16 years was approved in 2005). Therefore, this study could not infer directly on the impact of recent TCP on prevalence and inequalities. However, it was still possible to investigate the evolution of prevalence and inequalities in smoking from 2005 to 2014. Further studies could measure whether the legislation has been equally enforced across all the Portuguese municipalities and across different settings.

## 5. Conclusions

Among Portuguese men, inequalities in smoking against the worse-off have increased, while among women the gap reduced, but at the cost of an increase in the prevalence among the low-educated. From 2005 to 2014, there was a large reduction in SHS, and a significant reduction in daily smoking among men but not among women. SHS reduction was more marked among less privileged people. Future policies should be more comprehensive, to tackle simultaneously smoking and SHS prevalence, and their socioeconomic patterning. For example, comprehensive national (non-partial) bans, combined with price increases showed a consistent positive impact on inequalities [19].

## Figures and Tables

**Table 1:** Description of the sample.

	2005/06		2014		χ^2^ Test	
	N	Frequency	N	Frequency	*p*-Value	
**Sex**						
Men	13426	47.22	6679	43.95	<0.001	
Women	15007	52.78	8517	56.05		
**Age**						
25–44	10482	36.87	5031	33.11	<0.001	
45–64	11026	38.78	6054	39.84		
65–79	6925	24.36	4111	27.05		
**Education**						
Tertiary	2781	9.79	2491	16.39	<0.001	
Secondary	2882	10.14	2384	15.69		
Primary	3225	11.35	2372	15.61		
Primary first	15311	53.88	6433	42.33		
No education	4220	14.85	1516	9.98		
**Income**						
5th quintile	5906	21.17	3109	20.46	<0.001	
4th quintile	5426	19.45	3027	19.92		
3rd quintile	5401	19.36	2990	19.68		
2nd quintile	4493	16.10	2965	19.51		
1st quintile ^(a)^	6674	23.92	3105	20.43		

^(a)^ Lowest quintile of income. The income was provided in quintiles, for a question of data protection. So, the authors did not access information on individual income. Therefore, the quintiles may not be representing each 20%, when we excluded some age categories.

**Table 2:** Prevalence of smoking and SHS by sex, age, education, and income.

	Daily Smoking								Second-Hand smoking							
	2005-06 Sample			2014 Sample			Prevalence ratio ^(a)^ [IC95%]		2005-06 Sample			2014 Sample			Prevalence ratio ^(a)^ [IC95%]	
	n	Frequency	[IC95%]	n	Frequency	[IC95%]			n	Frequency	[IC95%]	n	Frequency	[IC95%]		
Total male	3978	27.16	[25.75;28.62]	1767	25.76	[24.20;27.39]	0.95	[0.87;1.03]	10,168	75.03	[73.60;76.40]	3368	53.77	[51.96;55.57]	**0.72**	[0.69;0.74]
**Age**																
25–44	2056	37.62	[35.10;40.20]	794	31.18	[28.43;34.06]	**0.82**	[0.74;0.93]	4255	83.24	[81.19;85.11]	1430	65.68	[62.73;68.15]	**0.79**	[0.75;0.83]
45–64	1515	25.48	[23.32;27.77]	815	28.41	[25.93;31.02]	1.11	[0.98;1.26]	4094	75.56	[73.29;77.78]	1391	51.29	[48.46;54.11]	**0.68**	[0.64;0.72]
65–79	407	11.46	[9.56;13.67]	158	9.15	[7.30;11.43]	0.79	[0.60;1.06]	1819	59.35	[56.00;62.61]	547	34.24	[30.93;37.72]	**0.58**	[0.51;0.65]
**Education**																
Tertiary	284	23.97	[19.83;28.67]	175	19.26	[15.77;23.33]	0.80	[0.61;1.05]	925	76.62	[71.80;80.83]	494	58.13	[53.40;62.72]	**0.76**	[0.68;0.84]
Secondary	457	30.59	[26.21;35.35]	299	25.65	[22.01;29.66]	0.84	[0.68;1.04]	1155	79.27	[754.95;83.02]	633	62.26	[57.87;66.45]	**0.79**	[0.72;0.86]
Primary	640	34.23	[30.04;38.68]	436	34.47	[30.60;38.56]	1.01	[0.84;1.20]	1344	80.85	[76.84;84.48]	720	60.80	[56.65;64.80]	**0.75**	[0.69;0.82]
Primary first	2282	26.84	[24.99;28.78]	771	24.79	[22.49;27.25]	0.92	[0.82;1.04]	5846	75.27	[73.38;77.06]	1379	46.60	[43.92;49.29]	**0.62**	[0.58;0.77]
No education	309	18.74	[15.07;23.05]	86	20.48	[14.92;27.39]	1.09	[0.75;1.59]	890	59.21	[54.25;63.98]	142	35.35	[28.66;42.65]	**0.60**	[0.48;0.64]
**Income**																
5th quintile	764	25.43	[22.52;28.56]	328	20.37	[17.57;23.49]	0.80	[0.66;1.97]	2217	73.23	[69.94;76.27]	842	56.60	[52.93;60.20]	**0.77**	[0.72;0.83]
4th quintile	813	28.78	[25.52;32.28]	339	23.40	[20.27;26.85]	**0.81**	[0.67;0.98]	2077	79.33	[76.20;82.14]	748	55.00	[51.19;58.75]	**0.69**	[0.64;0.75]
3rd quintile	757	26.26	[23.14;29.63]	364	27.64	[24.07;31.53]	1.05	[0.88;1.26]	1965	76.96	[73.67;79.94]	624	51.76	[47.63;55.88]	**0.67**	[0.61;0.74]
2nd quintile	706	31.45	[27.75;35.40]	330	29.59	[25.78;33.72]	0.94	[0.78;1.13]	1609	79.51	[76.16;82.50]	582	52.08	[47.85;56.28]	**0.66**	[0.60;0.72]
1st quintile (b)	856	25.16	[22.37;28.18]	406	30.61	[26.63;34.91]	**1.22**	[1.02;1.45]	2103	68.57	[65.38;71.59]	572	52.11	[47.54;56.63]	**0.76**	[0.69;0.84]
Total female	1381	9.42	[8.56;10.36]	984	11.79	[10.76;12.90]	**1.25**	[1.10;1.42]	7825	51.93	[50.40;53.44]	2967	38.29	[36.69;39.91]	**0.74**	[0.70;0.78]
**Age**																
25–44	954	17.84	[15.93;19.92]	519	17.216	[15.15;19.37]	0.96	[0.81;1.14]	3630	67.38	[64.89;69.77]	1401	53.60	[50.79;56.38]	**0.80**	[0.75;0.85]
45–64	389	7.38	[6.19;8.77]	413	11.89	[10.35;13.61]	**1.61**	[1.29;2.01]	3030	51.23	[48.79;53.66]	1146	34.68	[32.27;37.16]	**0.68**	[0.62;0.73]
65–79	38	1.11	[0.62;1.99]	52	2.43	[1.57;3.74]	**2.19**	[1.06;4.52]	1165	32.17	[29.48;34.98]	420	18.46	[16.17;20.99]	**0.57**	[0.49;0.67]
**Education**																
Tertiary	243	18.29	[14.99;22.13]	257	14.22	[11.80;17.04]	0.78	[0.59;1.02]	1131	68.65	[64.18;72.80]	778	51.54	[47.79;55.27]	**0.75**	[0.68;0.83]
Secondary	296	23.50	[19.39;28.19]	246	18.28	[15.39;21.56]	0.78	[0.60;1.00]	995	71.03	[66.30;75.35]	633	51.92	[47.89;55.93]	**0.73**	[0.66;0.81]
Primary	326	23.35	[19.47;27.73]	232	17.84	[14.94;21.16	**0.76**	[0.59;0.98]	1069	68.04	[63.43;72.32]	466	40.81	[36.65;45.11]	**0.60**	[0.53;0.68]
Primary first	487	5.24	[4.39;6.24]	225	7.00	[5.72;8.54]	**1.34**	[1.02;1.75]	3793	48.68	[46.57;50.79]	914	27.92	[25.66;30.29]	**0.57**	[0.52;0.63]
No education	27	0.83	[0.39;1.79]	24	3.05	[1.65;5.58]	**3.66**	[1.38;9.75]	834	31.65	[28.46;35.02]	176	17.94	[14.56;21.91]	**0.57**	[0.45;0.71]
**Income**																
5th quintile	435	16.08	[13.73;18.73]	221	12.44	[10.34;14.90]	**0.77**	[0.61;0.98]	1880	61.19	[57.81;64.47]	682	46.62	[42.93;50.35]	**0.76**	[0.69;0.84]
4th quintile	256	9.89	[7.89;12.33]	204	13.25	[10.91;15.99]	1.34	[1.00;1.80]	1558	58.39	[54.85;61.85]	642	41.51	[37.90;45.22]	**0.71**	[0.64;0.79]
3rd quintile	251	8.36	[6.61;10.52]	193	11.57	[9.14;14.53]	1.38	[1.00;1.92]	1433	50.79	[47.21;54.37]	595	39.28	[35.66;43.03]	**0.77**	[0.69;0.87]
2nd quintile	212	9.54	[7.42;12.19]	150	10.22	[8.22;12.65]	1.07	[0.77;1.49]	1208	52.98	[49.18;56.74]	479	31.31	[28.12;34.70]	**0.59**	[0.52;0.67]
1st quintile ^(b)^	205	4.64	[3.54;6.09]	216	11.53	[9.51;13.90]	2.48	[1.78;3.46]	1577	40.03	[37.16;42.97]	569	32.83	[29.47;36.37]	0.82	[0.72;0.93]

^(a)^ Prevalence ratio is the ratio between the prevalence in 2014 and in 2005–06 (significant values in bold); ^(b)^ Lowest quintile of income.

**Table 3:** Age-adjusted prevalence ratio of daily smoking and SHS exposure, stratified by sex, socioeconomic variable and sample.

	Daily Smoking				Second-Hand Smoking			
	2005/06 Sample		2014 Sample		2005/06 Sample		2014 Sample	
**Men, Education**								
Tertiary ^(a)^	1.00		1.00		1.00		1.00	
Secondary	1.22	[0.96;1.54]	1.31	[1.03;1.68]	1.02	[0.95;1.11]	1.06	[0.95;1.18]
Primary	1.46	[1.17;1.82]	1.85	[1.48;2.33]	1.07	[0.99;1.15]	1.08	[0.97;1.19]
Primary first	1.33	[1.09;1.62]	1.66	[1.32;2.08]	1.03	[0.97;1.10]	0.94	[0.85;1.04]
No education	1.33	[1.00;1.75]	1.66	[1.16;2.38]	0.90	[0.81;1.00]	0.78	[0.63;0.97]
**Men, Income**								
5th quintile ^(a)^	1.00		1.00		1.00		1.00	
4th quintile	1.17	[0.99;1.37]	1.20	[0.98;1.47]	1.09	[1.03;1.16]	1.00	[0.91;1.09]
3rd quintile	1.07	[0.90;1.26]	1.37	[1.13;1.67]	1.06	[1.00;1.13]	0.93	[0.84;1.02]
2nd quintile	1.20	[1.02;1.42]	1.58	[1.30;1.93]	1.08	[1.02;1.15]	0.98	[0.88;1.08]
1st quintile ^(b)^	1.16	[0.98;1.36]	1.56	[1.28;1.90]	0.99	[0.93;1.05]	0.95	[0.85;1.06]
**Women, Education**								
Tertiary ^(a)^	1.00		1.00		1.00		1.00	
Secondary	1.22	[0.93;1.60]	1.28	[0.99;1.64]	1.01	[0.93;1.11]	1.01	[0.91;1.12]
Primary	1.34	[1.03;1.74]	1.34	[1.04;1.73]	1.01	[0.92;1.11]	0.85	[0.74;0.96]
Primary first	0.38	[0.29;0.50]	0.66	[0.48;0.90]	0.79	[0.73;0.86]	0.69	[0.61;0.78]
No education	0.12	[0.05;0.28]	0.55	[0.28;1.08]	0.64	[0.56;0.73]	0.59	[0.46;0.75]
**Women, Income**								
5th quintile ^(a)^	1.00		1.00		1.00		1.00	
4th quintile	0.68	[0.52;0.89]	1.04	[0.80;1.36]	0.99	[0.92;1.08]	0.88	[0.78;0.99]
3rd quintile	0.57	[0.43;0.75]	0.98	[0.73;1.32]	0.86	[0.79;0.94]	0.87	[0.78;0.98]
2nd quintile	0.63	[0.47;0.83]	0.96	[0.73;1.28]	0.89	[0.82;0.97]	0.75	[0.66;0.85]
1st quintile ^(b)^	0.41	[0.30;0.55]	1.01	[0.78;1.32]	0.74	[0.68;0.81]	0.76	[0.67;0.86]

^(a)^ Reference category. ^(b)^ Lowest quintile of income. Note: Authors did not access information on individual income, thus the quintiles may not be representing 20% since we excluded some age categories.

**Table 4:** RII from the age-adjustedPoisson regressions, stratified by sex and sample.

	Daily Smoking						Second-Hand Smoking					
	2005/06 Sample		2014 Sample		Pooled Sample		2005/06 Sample		2014 Sample		Pooled Sample	
**Men, Education**												
RII	1.23	[1.00;1.52]	1.74	[1.36;2.22]	1.23	[1.00;1.51]	0.95	[0.88;1.02]	0.83	[0.73;0.95]	0.99	[0.92;1.07]
Survey year (yes = 2014)	-		-		0.79	[0.67;0.94]	-		-		0.80	[0.74;0.85]
RII x survey year ^(a)^	-		-		1.47	[1.09;1.99]	-		-		0.77	[0.67;0.89]
**Men, Income**												
RII	1.19	[0.98;1.43]	1.81	[1.45;2.24]	1.20	[1.00;1.46]	0.98	[0.92;1.06]	0.93	[0.83;1.05]	1.00	[0.93;1.07]
Survey year (yes = 2014)	-		-		0.75	[0.64;0.89]	-		-		0.74	[0.69;0.80]
RII x survey year ^(a)^	-		-		1.52	[1.14;2.02]	-		-		0.92	[0.80;1.06]
**Women, Education**												
RII	0.10	[0.06;0.15]	0.49	[0.32;0.75]	0.09	[0.06;0.13]	0.57	[0.50;0.64]	0.51	[0.43;0.62]	0.61	[0.54;0.68]
Survey year (yes = 2014)	-		-		0.63	[0.51;0.78]	-		-		0.78	[0.72;0.85]
RII x survey year ^(a)^	-		-		6.18	[3.71;10.32]	-		-		0.76	[0.63;0.92]
**Women, Income**												
RII	0.34	[0.24;0.50]	0.98	[0.72;1.33]	0.33	[0.23;0.48]	0.69	[0.62;0.76]	0.70	[0.60;0.81]	0.70	[0.63;0.78]
Survey year (yes = 2014)	-		-		0.74	[0.58;0.94]	-		-		0.73	[0.66;0.80]
RII x survey year ^(a)^	-		-		3.02	[1.89;4.83]	-		-		0.97	[0.81;1.16]

^(a)^ Interaction between Relative Inequality Index (RII) and sample.
